# When synthetic biology meets medicine

**DOI:** 10.1093/lifemedi/lnae010

**Published:** 2024-03-06

**Authors:** Yuge Feng, Cong Su, Guobin Mao, Baoting Sun, Yizhi Cai, Junbiao Dai, Yingxin Ma

**Affiliations:** CAS Key Laboratory of Quantitative Engineering Biology, Guangdong Provincial Key Laboratory of Synthetic Genomics and Shenzhen Key Laboratory of Synthetic Genomics, Shenzhen Institute of Synthetic Biology, Shenzhen Institutes of Advanced Technology, Chinese Academy of Sciences, Shenzhen 518055, China; Department of Biochemistry and Molecular Biology, College of Life Sciences, University of Chinese Academy of Sciences, Beijing 100049, China; CAS Key Laboratory of Quantitative Engineering Biology, Guangdong Provincial Key Laboratory of Synthetic Genomics and Shenzhen Key Laboratory of Synthetic Genomics, Shenzhen Institute of Synthetic Biology, Shenzhen Institutes of Advanced Technology, Chinese Academy of Sciences, Shenzhen 518055, China; CAS Key Laboratory of Quantitative Engineering Biology, Guangdong Provincial Key Laboratory of Synthetic Genomics and Shenzhen Key Laboratory of Synthetic Genomics, Shenzhen Institute of Synthetic Biology, Shenzhen Institutes of Advanced Technology, Chinese Academy of Sciences, Shenzhen 518055, China; CAS Key Laboratory of Quantitative Engineering Biology, Guangdong Provincial Key Laboratory of Synthetic Genomics and Shenzhen Key Laboratory of Synthetic Genomics, Shenzhen Institute of Synthetic Biology, Shenzhen Institutes of Advanced Technology, Chinese Academy of Sciences, Shenzhen 518055, China; Manchester Institute of Biotechnology, Department of Chemistry, University of Manchester, Manchester M17DN, United Kingdom; CAS Key Laboratory of Quantitative Engineering Biology, Guangdong Provincial Key Laboratory of Synthetic Genomics and Shenzhen Key Laboratory of Synthetic Genomics, Shenzhen Institute of Synthetic Biology, Shenzhen Institutes of Advanced Technology, Chinese Academy of Sciences, Shenzhen 518055, China; Department of Biochemistry and Molecular Biology, College of Life Sciences, University of Chinese Academy of Sciences, Beijing 100049, China; Shenzhen Branch, Guangdong Laboratory for Lingnan Modern Agriculture, Key Laboratory of Synthetic Biology, Ministry of Agriculture and Rural Affairs, Agricultural Genomics Institute at Shenzhen, Chinese Academy of Agricultural Sciences, Shenzhen 518000, China; CAS Key Laboratory of Quantitative Engineering Biology, Guangdong Provincial Key Laboratory of Synthetic Genomics and Shenzhen Key Laboratory of Synthetic Genomics, Shenzhen Institute of Synthetic Biology, Shenzhen Institutes of Advanced Technology, Chinese Academy of Sciences, Shenzhen 518055, China; Department of Biochemistry and Molecular Biology, College of Life Sciences, University of Chinese Academy of Sciences, Beijing 100049, China

**Keywords:** synthetic biology, medicine, disease diagnosis, disease treatment, metabolic engineering

## Abstract

In recent years, the world has faced significant challenges with the coronavirus disease 2019 (COVID-19) pandemic, as well as other infectious diseases such as Zika and Ebola. Furthermore, the rapid rise of non-communicable diseases such as diabetes, heart disease, and cancer has placed tremendous strain on healthcare resources and systems. Unfortunately, advancements in drug development, diagnostics, and therapeutics have struggled to keep pace with the emergence and progression of diseases, necessitating the exploration of new technologies for the discovery and development of biomedicines and biotherapies. Synthetic biology, a revolutionary field in modern science, holds great promise in advancing drug development and disease treatment. This review provides a comprehensive overview of recent developments in the application of synthetic biology to medicine, with a specific focus on its role in drug discovery, drug production, and the diagnosis and treatment of various diseases.

## Introduction

The coronavirus disease 2019 (COVID-19) pandemic has served as a stark reminder of the rapid and easy spread of diseases across borders, impacting populations worldwide. In recent years, other epidemics, including the Zika virus, Ebola, and SARS, have also demonstrated the alarming speed and scale at which infectious diseases can spread globally. Additionally, the rapid rise of non-communicable diseases (NCDs), such as diabetes, heart disease, and cancer, has placed tremendous strain on healthcare resources and systems. Unfortunately, advancements in drug development, diagnostics, and therapeutics continue to lag behind the rate of disease emergence and progression. Moreover, traditional clinical interventions have often prioritized treating patients after disease onset rather than implementing prevention or early intervention measures. Although several approaches have facilitated the discovery and development of contributing biomedicines and biotherapies, many pressing clinical needs remain unaddressed. Notably, a significant challenge lies in the lack of specificity and drug resistance. Therefore, there is an urgent need for new dynamic strategies to respond to emerging threats, improve the efficiency and effectiveness of existing treatments and therapies, and develop new personalized and targeted approaches to healthcare.

Synthetic biology is recognized as one of the ground-breaking technologies in modern science. It focuses on designing artificially engineered biological systems for specific purposes, utilizing modular systems ranging from simple units such as enzymes and regulatory elements, to complex modules built from simple units through precise tuning and mathematical combinations to form multiple genetic circuits. The potential applications of synthetic biology are vast, spanning from healthcare and medicine to agriculture and environmental protection. For example, in agriculture, synthetic biology can be used to develop new crops that are more resistant to pests and diseases or capable of producing higher yields. It also holds promise in the development of new biofuels and other sustainable sources of energy. In recent years, several significant achievements have been made in synthetic biology, such as the production of glucose and fatty acids from CO_2_ using a hybrid electro-biosystem [1], and the complete biosynthesis of cannabionoids and their unnatural analogs in yeast [2].

**Figure 1.**
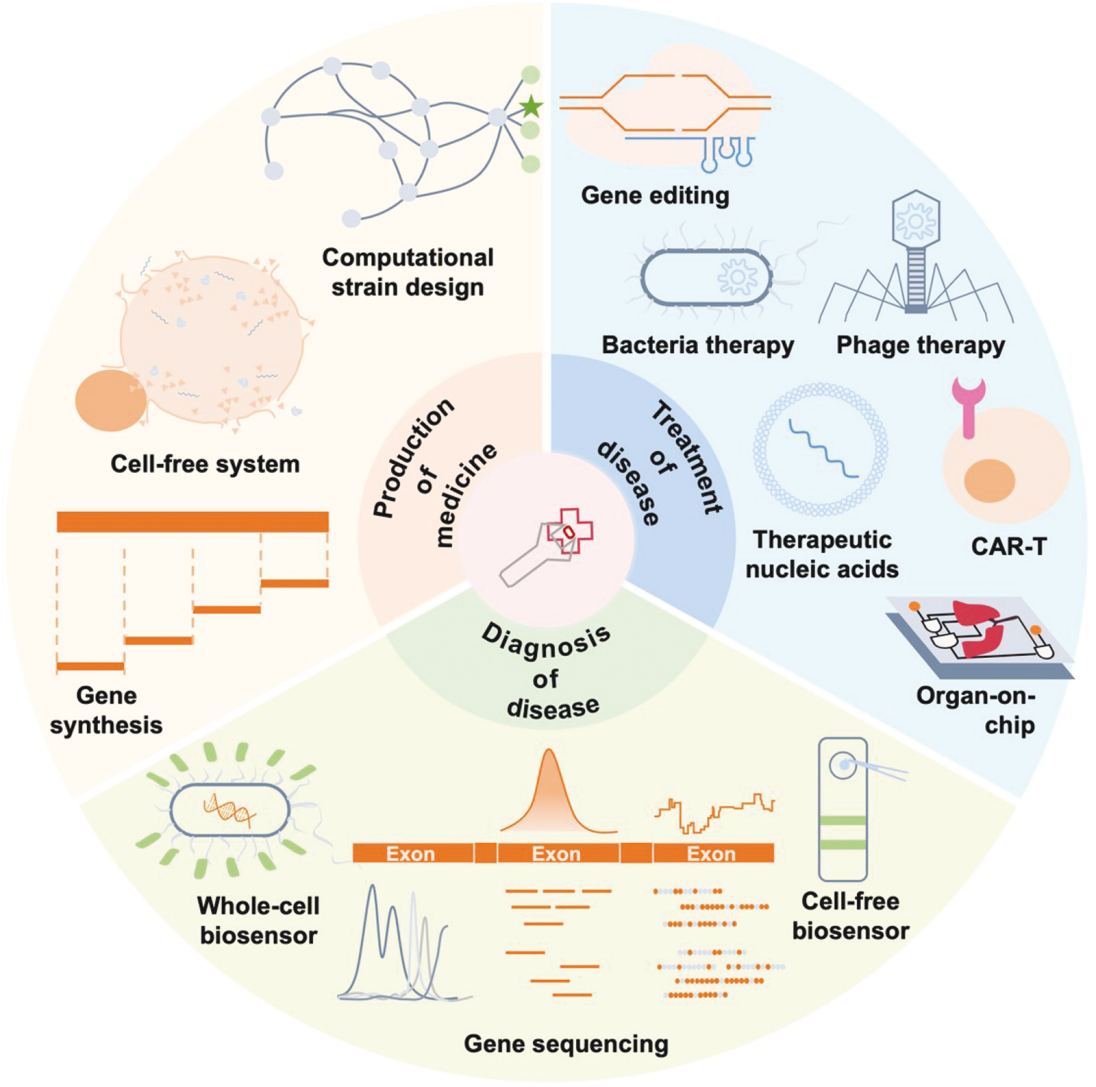
**When synthetic biology meets medicine.**Various synthetic biology methods and tools have been developed, and these new concepts are reforming the medical applications. Three axes of synthetic biology meeting medicine are summarized in this figure, show the acceleration of medical molecule production, the facilitation of disease diagnosis and revolution of disease treatment, respectively.

In this review, we focus on the applications of synthetic biology in the field of medicine, including drug discovery, drug production, and the diagnosis and treatment of various diseases (Fig. 1). Importantly, the modularity of synthetic biology allows for the deconstruction of designed frameworks into a series of interconnected composite operations. This modularity can be expressed as inputs and outputs in a broad sense, facilitating the implementation of various components, including proteins, nucleic acids, genome editing, and gene circuits. Moreover, this flexibility extends to cell-based or organ-on-chip systems, which can be engineered using synthetic biology to create more realistic models for drug discovery and disease research. Given the limitations of article length, many important studies may not be covered in this review. For those interested in exploring further applications of synthetic biology in medicine, we recommend referring to several other insightful reviews [3, 4]. Given the inherently interdisciplinary nature of synthetic biology, the concluding section discusses new technologies and trends in the biomedical field that may affect future research in synthetic biology.

## Synthetic biology accelerates the production of medical molecules

### General pipeline for drug production

The process of manufacturing drug molecules using synthetic biology involves several key steps: manipulation and generation of chassis cells, construction, and integration of molecular synthesis pathways, and artificial regulation and enhancement of metabolic networks. Through meticulous modifications, such as the deletion of large segments, proficient traceless editing, and stable expression of functional modules, interference from native background metabolites can be eliminated, or allogeneic expression can be enhanced. This optimization process improves the performance of chassis cells and augments the versatility of the “cell factory.” The production of a diverse range of target chemicals can be achieved through the manipulation of metabolic pathways, including optimizing precursor availability, regulating metabolic flow, and cofactor distribution at each reaction step within the metabolic network, and genetically modifying feedback inhibition mechanisms associated with metabolic intermediates and metabolites.

**Figure 2.**
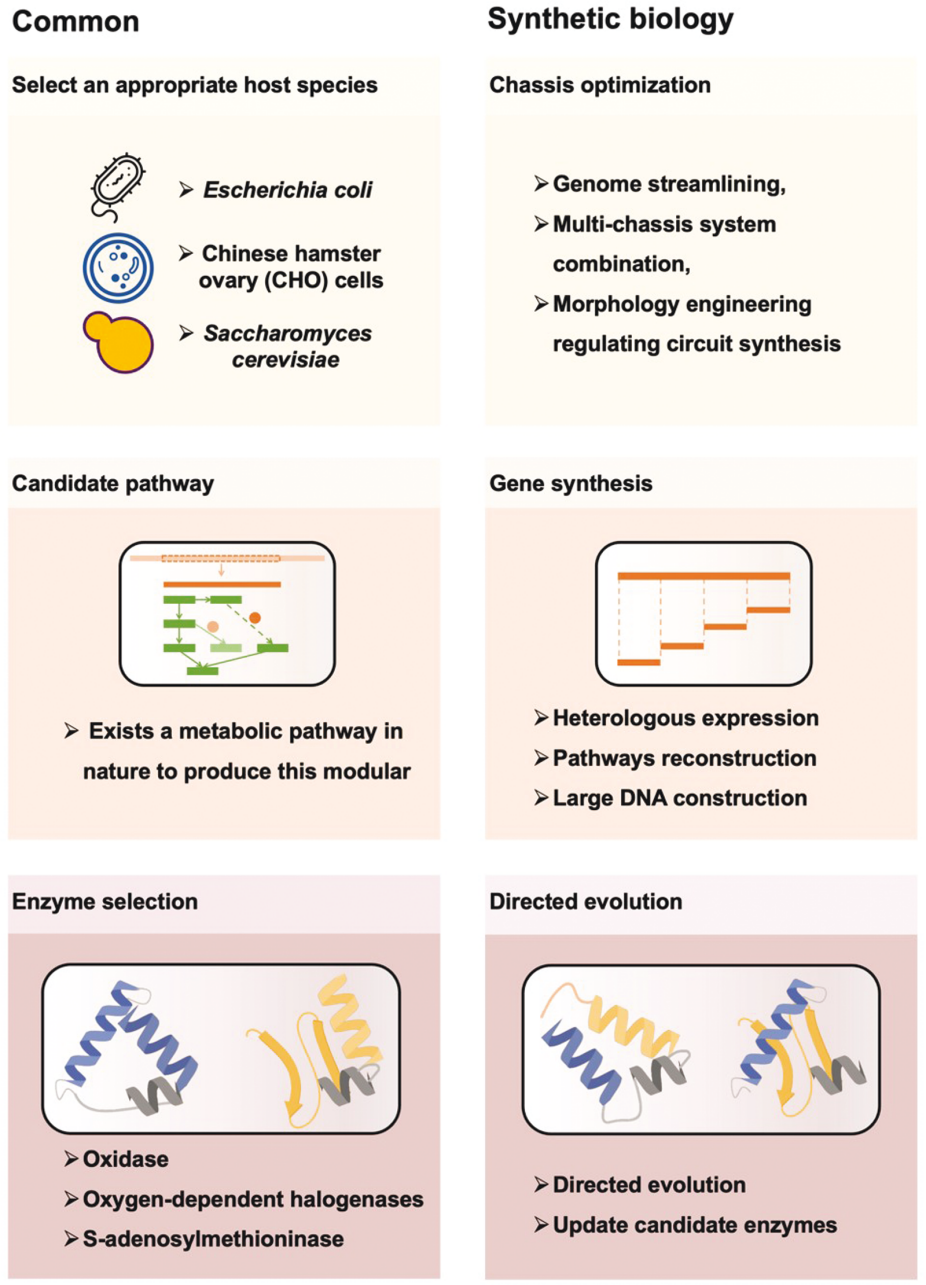
**General pipeline for synthetic biology drug production.**Common biopharmaceutical production (left) usually relies on natural hosts, pathways and enzymes. The drug molecules of such pipeline are extracted through fermentation of appropriate host heterologous expressed candidate pathway. The emergence of synthetic biology (right) and its potential for redesigning DNA and proteins within cells offer promising opportunities for drug molecular production. Furthermore, the test-build-design circle has facilitated rapid and extensive genetic manipulations. Colored circles illustrate test-build-design throughput.

For the production of a specific medical molecule in a cell factory, the first step is selecting an appropriate host organism (Fig. 2). The suitability of a host organism depends on several factors, including its genetic manipulability, growth rate, stability, and ability to produce desired products [5]. Host organisms that meet these criteria are often referred to as “manipulable engineered chassis” [6]. Microbial hosts are commonly referred, due to their well-developed techniques for genetic manipulation, cloning, culturing, and industrial scale-up. Prominent examples include *Escherichia coli*, *Bacillus subtilis*, *Corynebacterium glutamicum*, *Pichia. Pasteuris*, and *Saccharomyces cerevisiae*, which are relatively easy to handle and can grow in large quantities. Once the host is identified, the next important aspect is chassis optimization, which significantly expedites the development schedule of new products and technologies. By optimizing the chassis or host organism, researchers can improve the efficiency, specificity, and stability of an engineered system.

Various rapidly developed techniques are available for engineering the chassis cells, such as BAC-based transformation and expression, CRISPR-Cas9, transformation-associated recombination (TAR) cloning, and RecET direct cloning [7–10]. Ongoing efforts, including genome streamlining [11], multi-chassis system combination [12], morphology engineering [13], and regulating circuit synthesis [14] have also been implemented to facilitate the biosynthesis of complex small molecules. Other advances are under development, such as building artificial cells with minimized-genome as more controllable chassis [15] or incorporating non-standard amino acids (ncAAs) into recoded genome of protein drugs [16]. As a result, these synthetic biology strategies have provided unique insights into chassis optimization, subsequently accelerating the development of new drugs and medicinal molecules.

Pathway design is a key factor in constructing the entire synthesis system to produce small molecules of interest in an exogenous host. Two guiding principles are the atomic economy principle and the energy optimal principle. As the cost of oligo and gene synthesis decreases, researchers are increasingly venturing into the synthesis of compounds with longer metabolic pathways and higher levels of complexity, which accelerates the development of cell factories. Notably, the recent *de novo* synthesis of opioids [17] and cannabinoids [18] has shown significant potential for building cellular factories. When reconstructing complex and lengthy pathways, the use of multiple-module engineering helps divide pathways into smaller modules based on metabolic nodes [19] and functional clustering of components [20], which reduces pathway complexity. By establishing and optimizing module functions [21], adjusting module sub-organelle positioning [22], and regulating the expression intensity between modules [19], the combination design and iterative adaptation of modules have been successfully achieved. These technologies have been applied to produce the drug resveratrol, found in grape skin, and the antioxidant naringin, known for its anti-inflammatory and immune-stimulating effects. Although the supply of alternative drug-related compounds faces challenges, this success represents significant benefits of metabolic pathway engineering [23].

The assembly of these metabolic pathways necessitates specific enzyme production lines, including oxidase, oxygen-dependent halogenase, and S-adenosylmethionine enzymes, which serve as important functional units for constructing biocatalytic systems. However, accurately predicting sequence-function relationships remains a challenge. Hence, the tools of synthetic biology are essential to discovering new enzymes in nature on a large scale. Related research can provide high-quality parts for the development of industrial enzyme preparations and the construction of cellular synthetic metabolism. For example, Galanie et al. engineered yeast for opioid biosynthesis, achieving the optimization of a 23-enzyme pathway [24]. Another example is the production of milbemycins through a chimeric biosynthesis pathway of hybrid polyketide synthase, achieved through module replacement, demonstrating that this strategy, similar to advanced cell factories, will further increase the production of new natural compounds [25–27]. Protein engineering can be utilized to expand the substrate range of existing enzymes to accept larger substrates, as demonstrated by the production of monoterpene indole alkaloids (MIAs). Furthermore, the use of the design-build-test-learn (DBTL) cycle, involving the design, construction, and retesting of strains, can significantly accelerate the development of cell factories. Additionally, with the progress of DNA synthesis and automation, the development of new cell factories can be realized to a large extent through automated construction and testing procedures. The application of these technologies can greatly reduce the time and cost required for the development of cell factories [28].

### Common systems/chassis

Microorganisms such as *E. coli* and *S. cerevisiae* have been extensively studied and various resources are available, making them the most commonly used chassis for many products [29]. Other “platform cell factories” include *Aspergillus niger*, *Bacillus. subtills*, *C. glutamicum*, and Chinese hamster ovary (CHO) cells [30], each with specific advantages. For example, *S. cerevisiae*, being a eukaryotic chassis, offers ease of genomic integration due to a high rate of homologous recombination [31]. Conversely, *E. coli* boasts a doubling time, i.e. 3–4 times shorter than *S. cerevisiae*, making it well-suited for very high enzyme expression and having a different profile of native metabolites. *A. niger* and *B. subtills*, on the other hand, possess highly efficient protein secretion systems, making them popular choices for industrial enzyme production. Meanwhile, CHO cells are particularly well-suited for producing glycosylated proteins-focused pharmaceutical applications. Moreover, studies have reported successful production enhancements of specific chemicals using multiple organisms as co-cultures [23, 32, 33].

There are several success stories in pharmaceutical production utilizing different cell factories. A classic example is the production of artemisinin, an anti-malarial drug. It is first produced in *E. coli* [31] as amorphadiene, the precursor of artemisinin, and subsequently converted to artemisic acid, the oxidation product of amorphadiene, by introducing the eukaryotic cytochrome P450 enzyme CYP71AV1. However, the production of artemisic acid was initially limited, reaching only up to 10 g/L in *E. coli* [34]. By switching the chassis to *S. cerevisiae* S288C and later CEN.PK, researchers were able to boost the yield of artemisic acid to 25 g/L [35]. Another example is paclitaxel, which has shown promising therapeutic activity in breast cancer. It was demonstrated that its key intermediate, taxadiene, can be synthesized more efficiently in *E. coli* [36] than in *S. cerevisiae* [37]. Table 1 presents some examples of pharmaceuticals currently produced using engineered chassis.

**Table 1.** Common chassis and corresponding advantage applications

Type	Common chassis	Advantages	Common pharmaceutical application	Reference
*In vivo*	*E. coli*	Shorter doubling time, high expression of enzymes	Taxadiene	[36]
	*S. cerevisiae*	Ease of genomic integration	Artemisinin	[31]
	CHO	Well suited for production of glycosylated proteins	Glycosylated proteins	[30]
	*B. subtills*	Very efficient protein secretion	Bacillomycin	[28]
*In vitro*	Cell-free	Complexity of living organisms and bioactive molecule overproduction to the host	Oxytetracycline	[38]
	Whole-cell biocatalysis	It is easier to realize the *in situ* regeneration of energy and coenzymes	Glutathione	[39]

### In vitro/cell-free systems

While living cells dominate most biopharmaceutical production, cell-based systems producing modified proteins with biologically active and post-translationally modified modular formats are often limited to more complex intracellular protein manipulation. In contrast, the advanced synthetic biology tool, cell-free systems, is not limited by these constraints, and its role in biopharmaceutical product discovery and development continues to expand. These systems involve expressing all the enzymes required for a complete product synthesis pathway in host bacteria, and crude lysates or purified enzymes are mixed to reconstruct the entire metabolic pathway *in vitro* [40]. The construction of a cell-free biosynthetic system is characterized by strong robustness and expansibility.

Cell-free systems offer great flexibility in precisely controlling polypeptides without cell and membrane constraints, and they concentrate the use of energy without the need for highly evolved internal systems. These systems are based on natural cell extracts or purified protein synthesis components. By adding synthetic reaction substrates (DNA or mRNA templates, amino acids, nucleotides, etc.), energy substances, and necessary cofactors, the intracellular protein synthesis reaction is simplified into an extracellular solution reaction, achieving standardization of the reaction process. With the addition of template genes, gene transcription, translation, and even biological metabolism can be directly regulated and manipulated in an open environment.

Next-generation therapeutics aim to incorporate ncAAs into pharmaceutical products, and cell-free systems have demonstrated great potential for achieving this goal. These biopharmaceuticals require orthogonal components like transfer ribonucleic acids (tRNAs) and aminoacyl tRNA synthetases, which can be precisely optimized in cell-free systems, whereas their optimization in living cells is challenging due to transportation and compatibility issues. Cell-free systems have emerged as a novel enabling platform for the rapid prototyping of biosynthetic pathways and genetic circuits. Leveraging the rapid prototyping technology of cell-free protein synthesis systems, a complete cycle of “design-build-test-learn” for biosynthesis modules can now be accomplished in just a few days, significantly accelerating the development and application of these modules.

Over the years, through strain, protein, and platform engineering, cell-free systems expanded their scope to include the production of various biopharmaceuticals, such as antibody derivatives, antibody–drug conjugates, cytokines, vaccines, membrane proteins, metalloproteins, viral proteins, and antimicrobial peptide [41–45]. Bowie et al. modularized the 27-step synthesis pathway from glucose to limonene in *E. coli* [46], which involved central carbon metabolism, mevalerate acid modules, and a molecular purification valve module for coenzyme balance. After modular optimization, the conversion rate of limonene reached 88.3%. Subsequently, the research group attempted to convert glucose into limonene using crude lysates and successfully increased the yield to 3.8 mg/L per hour by establishing a modular limonene synthesis platform [47]. Cell-free systems offer impressive multi-faceted advantages. Grubbe et al. demonstrated better synthetic yields in a cell-free styrene biosynthesis platform compared to cell-based approaches, resulting in an increased titer from 5.36 ± 0.63 mM to 40.33 ± 1.03 mM [48]. Additionally, an on-chip cell-free research approach, known as Transcription-RNA Immobilization & Transfer-Translation (TRITT), has demonstrated further possibilities for automation, allowing for continuous and controllable protein production [49]. The successful synthesis of different antibody formats, including IgG, Fab fragments, and single-chain variable fragments (scFvs), has been achieved in different cell-free systems, such as in *E. coli* [44] and CHO [50], demonstrating the potential for handling complex products. Addressing some controversial points regarding cell-free systems, progress has been made in overcoming the inability to produce post-translationally modified glycosylated proteins. Bacterial glycosyl transferases have shown great promise in enabling this capability in *E. coli* cell-free systems [51]. These milestones indicate a promising future for on-demand production. Over the past decade, cell-free systems have evolved from being prototype methods in research labs to becoming commercially viable for large-scale production. Currently, more than 25 commercial cell-free systems are available for protein production in the market [52]. Moreover, products derived from cell-free systems based on *E. coli* lysates are already undergoing clinical trials.

These given productivity and controllability potential of cell-free systems, offer a promising platform for the key concepts of synthetic biology. These systems represent a shift in understanding biological mechanisms from a top-down perspective, allowing scientists to more precisely execute individual tasks from a bottom-up approach, which is the essence of synthetic biology. However, current modular “cell-free system” still face challenges in terms of upward integration and downward partitioning. For instance, cell-free systems are still not suitable for more complex synthetic processes, which demands significant optimization efforts, hindering the simplicity of these systems. Furthermore, toxic intermediates can inhibit certain enzyme activities in the overall reaction, requiring isolation and repair to protect them, as is the case in cells. The integration of cell-free systems with a variety of engineered systems is driving the next generation of synthetic biology. For instance, artificial intelligence have already begun to bring in more systematic design and mapping routes, and increasing stable proteins are being created through rational design. Beyond this, one can envision further partitions, such as “enzyme boxes,” which allow for precise matching of partners at the nanoscale and the interaction of various engineered enzymes. We anticipate that this field will continue to expand, taking synthetic biology to new horizons. We are excited to see the proteins no longer require challenging extraction, which is “game changing” for drug production and environmental conservation.

Here, it is worth briefly mentioning another extracorporeal strategy. Whole-cell bio-catalysis involves using complete biological organisms as catalysts for chemical transformation and utilizing intracellular enzymes for catalysis. This method lies between fermentation and extraction enzyme catalysis methods. Compared to microbial fermentation, whole-cell catalysis overcomes the shortcomings of long fermentation production cycles, complex metabolites, low substrate conversion, challenging product separation and extraction, and high energy consumption. In contrast to catalytic reactions using extracted enzymes, whole-cell catalysis enables the *in situ* regeneration of energy and coenzymes. The complete multienzyme system of cells allows for cascade reactions, compensating for the challenging enzymatic catalytic intermediate coupling process, improving catalytic efficiency, and reducing the need for enzyme purification, thus simplifying the preparation process and lowering production costs [39].

### Overview of representative drug products

The conventional approach to drug synthesis primarily involves chemical synthesis, which relies on costly chemical reagents, severe reaction conditions, intricate stereoselectivity, protracted synthesis pathways, and suboptimal overall yield. The emergence of synthetic biology has facilitated the artificial production of numerous drugs using biological cell factories, offering novel and supplementary avenues for total synthesis. In this section, we will provide a concise overview of the biosynthesis of various drug-active compounds at both laboratory and industrial scales (Table 2).

**Table 2.** List of pharmaceutical molecules produced in microorganisms

Product	Cell factory	Product application	Reference
Artemisinic acid	*S. cerevisiae*	Antimalarial drug	[31]
Taxadiene	*E. coli*	Anti-cancer drug	[36]
Cortisone	*S. cerevisiae*	Anti-inflammatory drug	[53]
Opioids	*S. cerevisiae*	Drugs to relieve pain	[32]
Reveratrol	*S. cerevisiae*	Antioxidant	[54, 55]
5-HTP	*E. coli*	Amino acid	[56]
Naringenin	*S. cerevisiae*	Anti-inflammatory, immune stimulating	[23]
β lactam antibiotics	*P. chrysogenum*	Anti-infective drug	[57]
Erythrocin	*S. erythraea*	Anti-infective drug	[58]
Tetracycline	*S. aureofaciens*	Anti-infective drug	[59]
Cyclosporin	*S. rosariensis*	Immune suppressing	[60]
Rapamycin	*S. rapamycinicus*	Immune suppressing	[61]
Lovastatin	*M. rubber*	Hypolipidemic drug	[62]

#### Lab scale

With the advancement of genomics, transcriptomics, proteomics, metabolomics, and bioinformatics, many anabolic pathways for medicinal molecules have been discovered. By reconstructing substance/energy metabolism and its regulatory pathways, synthetic biologists can achieve the total synthesis of drug molecules in the laboratory. Examples include paclitaxel, artificial unsaturated fatty acids, podophyllotoxin, cannabinoids, etc [35, 63–67]. Here, we will review the total synthesis of natural pharmaceutical molecules through homoeologous and heterologous microbial cell synthesis pathway reconfiguration.

##### Cannabinoids

In recent years, the biosynthesis of cannabinoids has been a groundbreaking advancement in synthetic biological manufacturing. Using *S. cerevisiae* as a chassis, Luo et al. optimized the synthesis pathway of substrate geranyl pyrophosphate (GPP) and introduced new cannabinoid synthetase and other engineering modifications [66]. By introducing the synthetic pathway of the cannabinoid precursor cannabinoid phenolic acid (CBGA) into yeast cells, they successfully achieved a breakthrough in the synthesis of CBGA in engineered microorganisms by replacing plant-derived isopentadiene transferase. Cannabinoids and their analogues were synthesized in a fermenter [66], and their artificial cultivation and extraction required 6 months, marking a significant milestone in synthetic biological manufacturing. This work lays the foundation for large-scale fermentation production of cannabinoids and provides an important reference for heterologous expression of other high-value-added natural drugs.

##### Monoterpene indole alkaloids

In the pursuit of enhancing and improving the drug potential of natural products, researchers have derived bioactive molecules with diverse structures and improved properties, potentially leading to new drugs. This is achieved through rational analysis of structure-activity relationships of natural products, mining synthetic and regulatory components, constructing a series of reaction modules and synthetic systems, and screening and optimizing chassis organisms.

For example, geranium pyrophosphate or amino acid base provides a starting point to potentially produce thousands of different plant natural products (PNPs). By producing platform strains of the key alkaloid (S)-reticuline [13, 14], scientists have facilitated microbes to generate papaver somniferum benzylisoquinoline alkaloids (BIAs) like morphine [68] and noscapine [69]. Strictosidine-producing strains [70] support biosynthesizing thousands of MIA compounds, including ajmalicine, vincristine and yohimbine.

##### Unnatural amino acids drugs

In addition to small molecule chemical drugs, the application of synthetic biology technology to alter the configuration of unnatural amino acids in protein or peptide drugs represents a promising avenue of investigation. Proteins in the body are typically made up of 20 naturally occurring amino acids, and different combinations and stereo conformations of these amino acids form the basis for the construction of a rich diversity of proteins. Breakthrough codon extension techniques developed in recent years enable the introduction of unnatural amino acids into target proteins, giving them new biological properties such as covalently binding neighboring proteins, carrying fluorescent groups, and simulating post-translational modification. At present, more than 200 unnatural amino acids have been introduced into proteins by gene codon extension technique [71, 72]. This technology holds significant translational value in the field of biological drug research and development. Compared with traditional small molecules, highly reactive covalent biologic drugs exhibit higher selectivity for target proteins and can significantly reduce off-target side effects [73, 74], opening up new opportunities for the design of biologic drugs with clear binding targets.

#### Industrial scale

While much of the research is still conducted in laboratories, many drug molecules are already being produced on an industrial scale in cellular factories. This approach partially addresses the issues associated with cumbersome chemical synthesis pathways, low yields, high energy consumption, heavy pollution, and challenges in achieving environmentally friendly large-scale production [19].

##### Amorphadiene

In 1986, Chinese scientist Xu et al. developed a chemical total synthesis method [75] to obtain artemisinin through multi-step chemical reactions. This method used r(+)-citronellol as a raw material, requiring a complicated multi-step reaction that led to unavoidable side reactions, resulting in a low yield of the final product, and limiting its industrial production. However, an alternative synthetic biology approach to produce artemisinin has been developed, involving the reconstruction of the artemisinin precursor pathway in microbial chassis cells, exogenous synthesis of artemisinin, and its synthesis through organic photochemical reactions. In a study on the synthetic biology of artemisinin, Keasling’s lab overexpressed all genes of the mevalonate pathway, inhibited the *ERG9* gene to enhance the squalene pathway for artemisinin synthesis, and expressed the artemisinin synthesis pathway exogenously, resulting in an artemisinin yield of 25 g/L, with the final product prepared with high purity through organic chemical reaction [35]. This technology is already being used by Thermo Fly for the industrial production of artemisinin.

##### Ginsenosides

A novel biosynthetic pathway of protoginseng diol in *S. cerevisiae* was constructed, along with the improvement of expression activity of key genes and optimization of the biphase fermentation process, increasing the yield of protoginseng diol to 1 g/L [76]. Subsequently, the first generation of “ginseng yeast” capable of simultaneously synthesizing oleanolic acid, proginsenediol, and proginsentriol was obtained [77]. Through molecular engineering, overexpression of key enzyme genes, and directed evolution, among other engineering strategies, ginsenoside yeast cells were constructed to improve the carbon supply of precursors and the conversion rate of target products, ultimately achieving a historical breakthrough of 179.3 mg/L in shake flasks and 2.25 g/L in batch fermentation [78]. The production capacity of “ginseng yeast” in a 1000 m^2^ workshop is equivalent to 100,000 mu (1   mu≈666.6667   m^2^) of ginseng cultivation, and the cost is 1/4 of that of ginseng cultivation and extraction.

## Synthetic biology facilitates disease diagnosis

### Sequencing technologies for identifying genes/pathogens related to genetic diseases

#### The importance of identifying genetic diseases/pathogens

Sequencing technology has played an indispensable role in the identification and treatment of endogenous diseases, particularly genetic disorders. The completion of the Human Genome Project has simplified the study of pathogenic gene mutations, leading to a significant increase in identifying pathogenic genes for rare monogenic diseases. Traditional diagnostic methods, such as imaging and biochemical testing, may not accurately diagnose all types of diseases, especially in the early stages or with atypical symptoms. However, DNA or RNA sequencing techniques can directly extract genetic material from patient samples, precisely identify diseases by analyzing genetic sequences, and assess disease risk based on genotype. For example, sequencing the *BRCA1* and *BRCA2* genes can screen high-risk groups for hereditary breast and ovarian cancer, enabling early diagnosis and prevention. Furthermore, sequencing the patient’s genome can reveal novel genes or gene variants associated with the disease, helping to elucidate the mechanisms of disease onset and progression. This enables individuals to choose the most effective treatment plan, receiving precision medicine and personalized treatment.

Sequencing technology also plays a crucial role in identifying exogenous diseases caused by pathogens. Traditional pathogen identification methods, such as microbial culture and immune testing, may not accurately identify all types of pathogens, especially in unknown or mixed infections. However, DNA or RNA sequencing technologies can directly extract genetic material from samples, precisely identify pathogens, and discover unknown pathogens. In these words, sequencing technology can swiftly detect and fully identify viruses, bacteria, fungi, and parasites without the need for prior pathogen inference. Pathogens derived from clinical samples exhibit heightened sensitivity and specificity, due to methods based on cultivation and separation. A prospective study on adults suffering from leukemia and febrile neutropenia suggests that sequencing results might allow 47% of patients to optimize antibiotics earlier [79]. Notably, in this study, a patient was detected with rhizobia at two different time points, and it took several days for fungal cultivation and identification. Another prospective study indicates that the sensitivity of sequencing technology is significantly higher than that of cultivation alone [80]. When detecting patients with significantly increased cell counts and protein levels, the chance of combining positive results with mycobacteria is higher. However, up to 60% of infectious disease causes remain unknown [81]. For emerging, rare, or challenging infectious diseases, sequencing technology offers advantages not found in traditional diagnostics. For instance, the first case of encephalitis caused by variegated squirrel bornavirus-1 (VSBV-1) was reported in 2015, and the first case of endophthalmitis caused by human-infecting porcine herpesvirus was reported in 2018 [82, 83].

Analyzing the genomic information of known pathogens can help us understand their genomic characteristics, such as drug sensitivity or resistance, and predict their evolution and mutation trends, which is conducive to formulating effective public health strategies. During the novel coronavirus pandemic, sequencing technology analyzed the phylogenetic structure of the novel coronavirus and predicted evolutionary mutation trends, playing a significant role in front-line diagnosis. Sequencing technology can also be used to identify key antigenic sites of pathogens, providing substantial support for vaccine design and development. Notably, due to the shortcomings of traditional pathogen identification, leading to the overuse of empirical broad-spectrum antibiotics or antifungal drugs, sequencing technology is less affected by prior exposure, demonstrating greater potential. In short, the sequencing technology has been proven to be an effective method for infectious disease diagnosis, and further development of sequencing technology will pioneer the integration of host and pathogen sequencing data, providing a more systematic diagnosis and advancing clinical management.

Therefore, sequencing technology plays a crucial role in genetic diseases caused by both internal factors and external pathogens. It effectively promotes early prevention and treatment of diseases by improving diagnostic accuracy, discovering new pathogens, and studying the evolution and mutation of pathogens. The changes brought about by this technology have deepened our understanding of diseases, provided a strong impetus for the progress of modern medicine, and had a profound impact on improving global public health standards.

#### Iteration of sequencing technology

Despite facing numerous challenges, the advent and ongoing innovation of sequencing technologies furnish powerful instruments for identifying exogenous and endogenous diseases. Each iteration of sequencing technology accelerates our comprehension of genetic mechanisms underlying diseases. Since its inception in 1977, Sanger sequencing [84], the pioneer of sequencing technology, has played a significant role in decoding numerous genomes [85–88]. Despite its high precision, the limitations of Sanger sequencing, such as low throughput, high cost, and extensive time requirements, have curtailed its utilization in disease and pathogen identification.

The advent of Next-Generation Sequencing (NGS) technology in 2005 significantly transformed this landscape. NGS revolutionized sequencing speed and throughput by enabling the parallel processing of multiple DNA samples, thus catalyzing further advancements in genomic research [89]. For example, whole genome sequencing (WGS) has been employed to investigate the relationship between human and animal genomes and diseases [90–92], while transcriptome and epigenome sequencing have been used to explore gene expression and regulation mechanisms [93, 94]. However, NGS does have certain limitations, such as short read length and amplification bias, which can pose challenges when dealing with complex genomic structures.

In response to these limitations, third-generation sequencing (TGS) technology, also known as single-molecule sequencing, emerged. These novel technologies can directly sequence individual DNA molecules without the need for PCR amplification, resulting in ultra-long read lengths that facilitate the resolution of complex genomes and repeated sequences. For instance, nanopore and PacBio sequencing technologies have been successfully applied in disease genomic analysis, revealing unknown pathogenic mechanisms and offering possibilities for new therapeutic strategies. For example, in 2017, the deployment of MinION and Illumina sequencing for the genomic detection of the Ebola outbreak in West Africa significantly augmented our comprehension of the disease [95]. Furthermore, in 2022, the reporting of a gapless human genome sequence marked the formal completion of the Human Genome Project, unveiling high homology repetitive segments and variations within the human genome [96–100]. These strides reaffirm the crucial role that TGS plays in diagnosing and treating diseases.

Despite the advantages of TGS over NGS and Sanger sequencing in certain aspects, its prohibitive cost hinders its full replacement of the preceding technologies. Continuous innovation in sequencing technology may lead to the advent of lower-cost, high-throughput, and single-molecule sequencing tools in the future, opening up more opportunities for disease discovery at the genetic level.

### Whole-cell and cell-free biosensors for health and environmental monitoring

Synthetic gene circuits refer to the rewiring of genetic expression elements, enabling specific patterns of gene expression to execute complex tasks. Benefic by their ability to perform complex tasks, such as streamlining processes and rapid iteration, gene circuits are often defined as biological sensors and combinations, i.e. molecular circuits that program complex cellular functions. In fact, this is a commonly used but narrow definition. Although in this review, we prefer to emphasize the extensive componentization idea of gene circuits. As pointed out at the beginning, this componentized framework enables a series of complex interactions for regulation, not only from genes to proteins, but also including gene-to-gene regulation and the supplementation of synthetic components, providing endless possibilities for re-create and manipulable synthetic biomedicine. The historical molecular tool set has been increasingly supplemented by modular and programmable biological molecules, which can connect different synthetic and natural systems, allowing for a more seamless integration of synthetic systems into natural systems [101]. However, it is undeniable that under the defined concepts of gene circuits as cell sensors, a series of creative medical applications have been generated, especially in the field of disease diagnosis. From traditional whole-cell activity identification to biosensors utilizing natural disease biology and re-established enzyme functions, more sophisticated clinical performance has been achieved, characterized by sensitivity, specificity, portability, and sustainability [4]. Here, we will mainly mention whole-cell biosensors and cell-free biosensors as the two most prominent synthetic biological detection devices currently, along with some typical examples and tools.

#### Whole-cell biosensor

Whole-cell biosensors are cells containing transducer elements that act as receptors to detect chemical changes or physiological pressures, converting recognition events into detectable signals. These cells have genetic elements or circuits that regulate the expression of reporting elements after the binding of analytes [102]. The basic genetic detection circuit comprises three types of components: sensing, signal processing, and output elements [103]. In this section, we will introduce the concepts and mechanisms of whole-cell biosensors, as well as their applications and strategies for improvement in human health monitoring.

**Figure 3.**
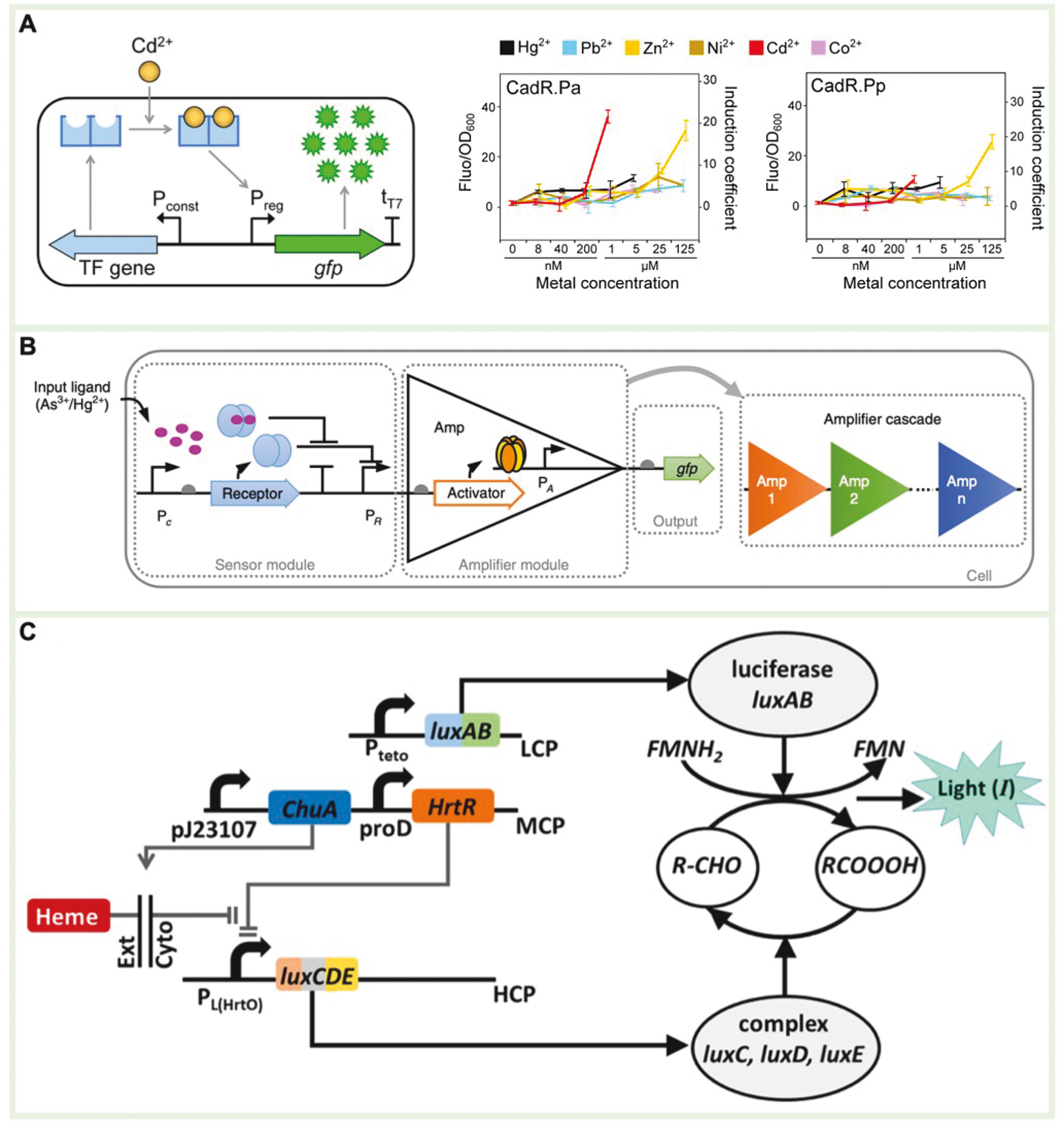
**Whole-cell biosensors.**(A) Cadmium ions bind to TFs and activate GFP expression [106]. Reproduced with permission [Ref. 94]. Copyright 2021, *ACS*. (B) Modular multi-layer signal amplification engineering for the ultra-sensitive transcriptional cell sensors [109]. Reproduced with permission [Ref. 110]. Copyright 2023, *Springer Nature*. (C) The re-engineered splitting *luxABCDE* reporting system for heme detection [102]. Reproduced with permission [Ref. 90]. Copyright 2021, *ACS.*

The sensing element of a whole-cell biosensor typically consists of a transcription factor (TF) used for analyte recognition and induction of downstream gene expression [104]. Various analytes recognized by TFs include heavy metals, quorum-sensing molecules, antibiotics, and disease-related biomarkers [105]. For example, He et al. screened 16 types of cadmium (Cd)-sensing TFs from the GenBank database, and expressed green fluorescent protein (GFP) as the output signal [106]. In the engineered *E. coli*, the system demonstrated efficient detection for 14 different metal ions with high specificity and a strong signal-to-noise ratio (Fig. 3A). To expand the TF library and identify more analytes, Jha et al. designed a novel Acinetobacter TF capable of recognizing p-nitrophenol (pNP) and inducing the expression of fluorescent protein to specifically detect the insecticide paraoxon [107]. This system utilized an independent promoter to express phosphotriesterase (PTE), which catalyzes the hydrolysis of paraoxon, producing pNP as the output signal. Another sensing element used in whole-cell biosensors is a two-component regulatory system, composed of a recognition protein and a response regulator, which is commonly used for the detection of extracellular events. For example, the recognition protein CusS binds to Cu ions, activating the response regulator and initiating the expression of riboflavin, resulting in electrical signal changes that allow for rapid and cost-effective *in situ* monitoring of Cu ions [108].

The signal processing element converts the sensing event into a measurable signal to achieve analyte detection [105]. These elements typically involve amplifiers, feedback loops, and/or logic gates to adjust the output/input signal ratio. Wan et al. designed a multi-layer transcription amplifier to improve the expression level of output signals for ultra-sensitive detection of arsenic and mercury ions [109]. The first-layer recognition module was sequentially connected to the second and third-layer amplifiers, increasing the detection limit and output signal by 5000 and 750 times, respectively (Fig. 3B). Luisi et al. developed an agentic amplification circuit to detect 2-phenyl phenol and produce a visual colorimetric output [110]. 2-Phenyl phenol induced the expression of HrpR and HrpS proteins, and reporter genes. The HrpR and HrpS proteins formed a complex that drove the expression of more HrpR and HrpS proteins, and reporter genes, resulting in a 66-fold increase in output signals. Woo et al. developed a Boolean AND gate-based biosensor for the simultaneous detection of thiosulfate and nitrate biomarkers [111]. Nitrate-induced phosphorylation of the NarXL protein, activating the expression of ThsS (L547T) R protein. Subsequently, thiosulfate drove the phosphorylation of the expressed ThsS (L547T) R protein, promoting the expression of the sfGFP reporter protein. The Boolean AND gate sensor could only respond when nitrate and thiosulfate coexisted, effectively reducing false positive results during the diagnostic process.

Many reporter proteins that generate fluorescence, color, electrons, or gas can serve as output elements. Recombinant DNA technology has significantly contributed to the construction of reporter protein libraries based on biological genetic coding. Lopreside et al. compared the effects of eight signals generated from three types of output elements including fluorescence (*gfpmut3*, *deGFP*, *mCherry*, *mScaret-I*), colorimetric (*lacZ*), and bioluminescence (*luxCDABE* from *Aliivibrio fischeri* or *Photorhabdus luminescens*, *NanoLuc*) in mercury and quorum sensing molecular detection [112]. The results showed that *NanoLuc* was the most sensitive reporter gene for detecting these two representative analytes. Barger et al. re-designed the genetic structure of the *luxCDABE* operon, splitting it into *luxCDE* and *luxAB* to improve the biological detection performance for heme [102]. The *luxCDE* gene was regulated by a heme-induced promoter, and the *luxAB* gene was driven by a constitutive promoter. As a result, the output signal could only be generated in the presence of heme, exhibiting a higher fold-change response and better specificity (Fig. 3C).

With the rapid development of genetic circuits and instrument-free measurement, whole-cell biosensors have evolved into a promising point-of-care testing platform for environmental monitoring and biomedical applications, offering numerous opportunities for human health and environmental protection. For example, integrating whole-cell biosensors into wearable clothing or living patches for real-time analysis of biological metabolites or combining them with autonomous robots or drones to monitor inaccessible environments represents a new frontier in the application of whole-cell biosensors.

#### Cell-free biosensor

A cell-free system refers to obtaining essential components for transcription and translation from cells and adding DNA templates to maintain gene transcription, protein translation, or metabolic processes in an *in vitro* system, thereby achieving the synthesis of target products [113]. The cell-free biosensor is a type of trace substance detection tool designed based on the cell-free system, which offers the advantages of eliminating transmembrane transport barriers and overcoming biosafety and nutritional limitations associated with cell storage. It has received widespread attention in rapid detection applications, including metal ions, quorum-sensing molecules, antibiotics, viruses, and other fields. However, it also brings new challenges, such as the lack of physical barriers to protect system components and the decrease in component activity during freezing-drying processes. This section introduces recent achievements in improving the sensitivity and portability of cell-free biosensors, as well as discusses future development trends in this field.

##### Transcription factor activation and repression

TF is a type of protein molecule that specifically binds to certain sequences upstream of the 5ʹ end of a gene, ensuring the precise and spatially controlled expression of the target gene at specific intensities and times [114]. The specificity of TFs makes them valuable as recognition components in biosensor design, where the expression of the reporter gene can be achieved through the binding of activated TFs to the detected ligand or the release of inhibitory TFs after ligand binding. The products of reporter gene expression mainly include proteins, fluorescent aptamers, and riboswitches, which are commonly monitored through fluorescence or colorimetric methods [115].

**Figure 4.**
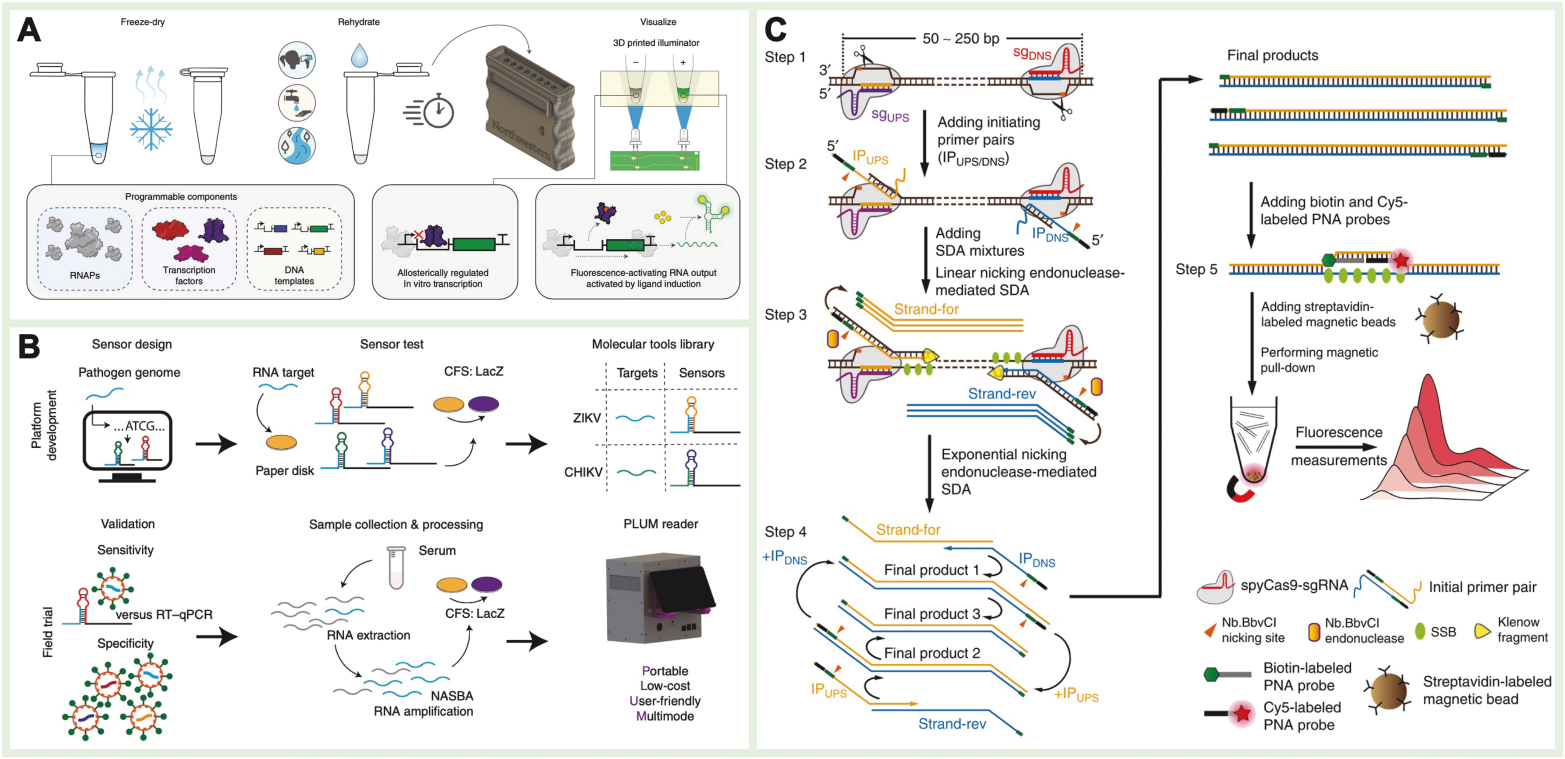
**Cell-free biosensors.**(A) The ROSALIND system for analyte of interest detection using a low-cost, 3D-printed handheld device [117]. Reproduced with permission [Ref. 97]. Copyright 2023, *Springer Nature*. (b) The paper-based toehold-switch diagnostic system for Zika and chikungunya viruses’ detection [119]. Reproduced with permission [Ref. 98]. Copyright 2022, *Springer Nature*. (C) A scheme showing the reaction mechanism of CRISPR-Cas9-triggered nicking endonuclease-mediated strand displacement amplification method for DNA detection [120]. Reproduced with permission [Ref. 109]. Copyright 2018, *Springer Nature.*

Recently, various TFs with specific binding capabilities to ligands such as heavy metals and antibiotics have been screened, and an induced RNA output sensor platform known as ROSALIND has been constructed for rapid ligand detection. Zhang et al. utilized surface plasmon resonance to screen TFs specifically bound to Hg^2+^ and Pb^2+^ for on-site detection of heavy metals in ambient water [116]. The strong affinity between heavy metal ions and TFs led to the release of inhibitory TFs from the TF binding site, enabling *in vitro* transcription to produce fluorescent RNA and achieving paper-based detection within 1 h. Jung et al. developed TF-based biosensors that, when combined with targets, induce the expression of fluorescent-activated aptamers for detecting pollutants such as antibiotics, small molecules, and metals in water [117]. By integrating 3D-printing technology, they obtained a low-cost handheld device for on-site water quality monitoring (Fig. 4A). TF-based biosensors heavily rely on the specific binding of TFs to ligands. However, only a limited number of analytes can be directly recognized as ligands or converted into recognizable ligands through metabolic enzyme catalysis using existing TFs [118]. To further expand the range of analytes and improve the specificity and sensitivity of TFs, a directed evolution strategy can be used for efficient TF screening.

##### Toehold switch

The toehold switch is a programmable nucleic acid switch consisting of two RNAs: switch RNA and trigger RNA [121]. The stem-ring structure of the switch RNA blocks its ribosome binding site (RBS), inhibiting the translation process, and when the trigger RNA complements with it, the RBS is released, relieving the inhibitory effect on gene expression. In a cell-free system, the target nucleic acid serves as the trigger RNA, activating the expression of the reporter gene and enabling high-precision detection of the target. Pardee et al. used isothermally amplified Zika virus RNA as the trigger RNA for a toehold switch on paper, which activated the expression of the *LacZ* gene and generated detection signals, creating a point-of-care test platform for use outside the laboratory [111]. By combining the CRISPR-Cas9 system, single-base resolution identification of virus strains can be achieved, facilitating rapid responses to global epidemics. Furthermore, Pardee et al. developed a low-cost, automated, and computer-vision plate reader for low-capacity and high-throughput target detection in a 384-well plate [115]. The detection sensitivity and specificity for Zika virus and chikungunya virus were comparable to real-time quantitative PCR (Fig. 4B) [119]. Currently, toehold-switch biosensors are primarily used for nucleic acid detection, and expanding their detection range to include small molecules and proteins remains a significant challenge.

##### CRISPR-Cas recognition

CRISPR-Cas-related biosensors are widely used in the detection of both nucleic acid and non-nucleic acid targets, relying on the specific recognition of CRISPR-RNA (crRNA) and the *trans*/*cis* cleavage of activated Cas proteins [122]. Various types of Cas proteins, such as Cas9, Cas12, and Cas13, are used in biosensor construction. The *cis*-cleavage activity of the Cas9 protein is activated after guide RNA binds with the target double-stranded DNA (dsDNA), resulting in the cleavage of the target sequence. The binding of crRNA to target DNA activates Cas12a, leading to double-stranded breaks at the crRNA binding site and non-specific cleavage of surrounding single-stranded DNA (ssDNA), thus achieving target DNA endo-nucleation and signal amplification. As a subtype of Cas13, Cas13a also exhibits *cis*- and *trans*-cleavage activity, recognizing single-stranded RNA (ssRNA) and cleaving target or non-target ssRNA. Zhou et al. identified target DNA through Cas9/sgRNA and induced conformational rearrangement to initiate nicking endonuclease-mediated strand displacement amplification (Fig. 4C), achieving attomolar sensitivity, and single-nucleotide specificity for DNA detection in complex samples [120]. Additionally, our group developed a recombinase polymerase amplification (RPA)-assisted CRISPR-Cas12a system, which utilized ALP-ssDNA modified magnetic beads as the reporting probe, enabling single-copy detection of African swine fever virus [116].

Non-nucleic acid targets, such as small molecules, proteins, and pathogens, are transduced into nucleic acid signals for CRISPR-Cas-related biosensors using aptamers or DNA-modified antibodies [123]. Shen et al. detected a very low number of *Salmonella Enteritidis* cells without isolation based on a combination of nucleic acid allosteric probes and CRISPR-Cas13a components [121]. Compared with traditional real-time PCR, this approach exhibited similar or higher sensitivity and accuracy. Tang et al. combined antibody-induced nucleic acid assembly and RPA technology to obtain target dsDNA, which, when combined with CRISPR-Cas12a, cleaved the ssDNA reporter to generate fluorescence signals [118]. The sensitivity of this method for detecting SRAS-CoV-2 spike protein antibody was 10,000 times higher than that of traditional ELISA. CRISPR-Cas-related biosensors are often combined with RPA, PCR, loop-mediated isothermal amplification (LAMP), hybridization chain reaction, and rolling circle amplification to achieve higher sensitivity [113]. However, these methods are susceptible to off-target effects and false positives, which remains a major challenge in this field.

## Synthetic biology revolutionizes disease treatment

**Figure 5.**
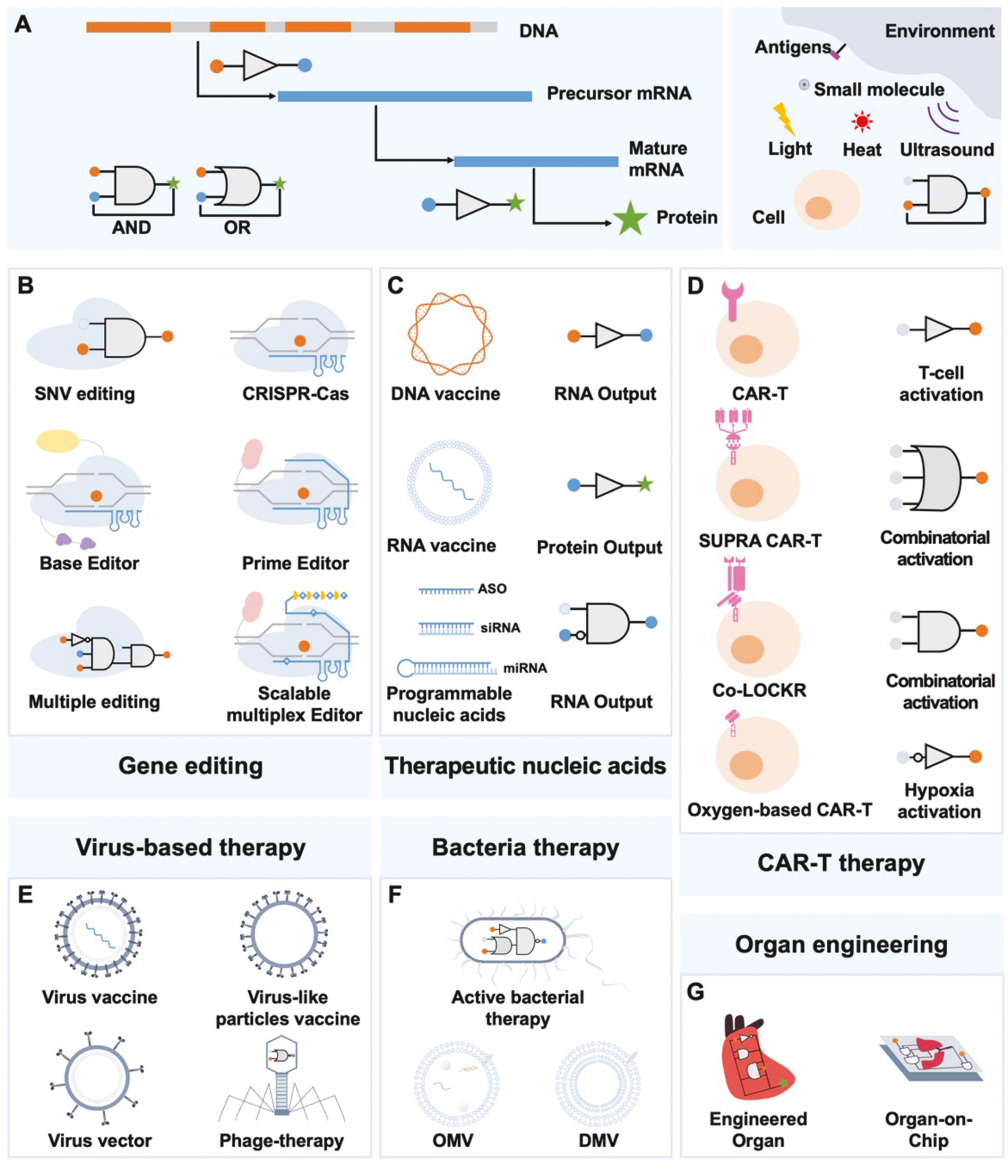
**Synthetic biology revolutionizes disease treatment.** (A) Modular operation in synthetic biology and their regulatory location. Engineered gates sense and respond to endogenous translation, transcriptional events, and exogenous stimuli, enabling control of both nucleic acids-based and cell-based therapy. (B) Gene editing. Simple gene editing tools require just one input, and have been applied to accurately target SNV. Such modules could be multiplied to cope with complex diseases. (B) Therapeutic nucleic acids. (D) CAR-T therapy. (E) Virus-based therapy. (F) Bacteria therapy. (G) Organ engineering. Orange node: DNA; blue node: RNA; green node: protein.

Disease treatment stands as the quintessential challenge in the realm of medicine. It entails two fundamental steps in addressing the human body’s innate response to ailments: the precise targeting of the afflicted site and the implementation of an efficacious treatment modality. Conventional chemical and physical therapies, while serving their purpose, may inadvertently inflict varying degrees of harm to healthy cells or be limited to localized intervention, consequently rendering disease eradication arduous. Numerous groundbreaking advancements in the fields of biochemistry, molecular biology, bioinformatics, and structural biology have yielded significant headway in terms of targeting and therapeutic efficacy. Nonetheless, the advent of synthetic biology has ushered in a complete transformation in the approach to disease treatment. Embodying a programmable design paradigm, this concept manifests across various stages and operates in response to diverse intracellular and exogenous signals (Fig. 5A). Moving forward, our focus converges on several pioneering strategies, which have been successfully applied to the treatment of a myriad of diseases. These approaches have garnered approval or are presently undergoing clinical trials.

### Treating genetic diseases by gene editing

Gene editing is one of the most powerful and widespread tools of synthetic biology, holding great potential for next-generation gene therapy. In this section, we will briefly review the progress of using gene editing tools to treat genetic diseases, with a primary focus on CRISPR-Cas, base editing, and primer editing. Each tool possesses its capabilities and limitations, and considerable efforts are being made to improve their efficiency in treating single-nucleotide variants (SNV) diseases and expanding their scope of treatment for more complex diseases (Fig 5B).

#### Treating diseases caused by single-nucleotide variants

Approximately 90% of known pathogenic genetic variants are attributed to SNVs [124], underscoring the importance of developing tools capable of efficiently correcting SNVs. One effective treatment strategy involves replacing the mutated sequence with the correct nucleotides, and progress has been made in using this approach to treat several human diseases, including thalassemia [125], cystic fibrosis [126], Parkinson’s disease [127], Huntington’s disease [128], and Duchenne muscular dystrophy (DMD) [129]. These diseases involve specific single nucleotide mutations in genes such as *HBB* [130], *CFTR* [131], *SNCA* [132], *HTT* [133], and *DMD* [134]. Recent advances in gene editing tools have provided new hope for effectively and safely treating these genetic diseases. In this section, we will focus on CRISPR-Cas, base editing, and primer editing, and compare the advantages of the latter two tools in treating DMD as a classical case.

The CRISPR-Cas9 system is derived from a naturally occurring system in bacterial and archaeal. CRISPR-Cas9 genome editing involves generating a Cas9-induced double-strand break that can be repaired by either non-homologous end joining (NHEJ) mechanisms or homology-directed repair (HDR). The designed gRNA targets the pathogenic DNA sequences to direct the Cas9 nuclease enzyme, which has shown great potential in treating SNV diseases. For example, β-thalassemia, a group of blood disorders resulting from *HBB* mutations, is considered one of the most common human monogenic inherited diseases [135]. Dever et al. devised a CRISPR-based gene editing system that combines Cas9 and adeno-associated viral vector delivery of a homologous donor, achieving targeted recombination at the *HBB* locus [136].

In contrast to CRISPR-Cas, base-editors function by directly modifying target single-nucleotides with precision, without generating double-strand breaks (DBSs). Therefore, they have been rapidly adopted in emerging human therapeutics. Two main classes of base editors have been developed: cytosine base editors (CBEs), which catalyze the conversation of C–G to T–A, and adenine base editors (ABEs), which catalyze A–T to G–C conversions [137]. Furthermore, over 25% of human pathogenic SNVs could be corrected by targeting the four transition mutations [138]. For example, DMD, a monogenic recessive neuromuscular disease, results in patients suffering from degeneration of cardiac and skeletal muscles [139], and it is caused by mutations in the *DMD* gene. Chemello et al. developed an ABE that modified splice donor sites of the dystrophin gene, resulting in the skipping of a prevalent *DMD* deletion mutation of exon 51 (Δex51) in cardiomyocytes derived from human induced pluripotent stem cells, restoring dystrophin expression. This restoration of dystrophin expression was also demonstrated in the mouse model using a split-intein dual-AAV system [140]. Geurts et al. described a cystic fibrosis intestinal organoid biobank representing 664 patients and 164 *CFTR* mutations, of which 20% could theoretically be repaired by ABE. The study showed efficient repair of nonsense mutations in *CFTR* without any detectable off-target effects [141].

Prime editors, fusion proteins between a Cas9-nickase domain and an engineered reverse transcriptase domain, do not require double-strand DNA breaks, making them more versatile and suitable for precise favorable editing [142]. Due to this versatility, prime editing extends the therapeutic scope beyond the direct correction of pathogenic mutations. As mentioned before, DMD commonly involves large deletions in exonic regions, resulting in frame-shifted transcripts [143]. Therefore, restoring the reading frame by deleting another exon has been an effective therapeutic approach for DMD. However, producing nearly full-length dystrophin protein could only partially rescue its function. Chemello et al. used Pes to insert 2 bp to restore the *DMD* expression, resulting in the deletion of Δex51, and through precise exon reframing, they minimized the amino acids missing from the final dystrophin protein [140]. Anzalone et al. examined twinPE with the same strategy for excising Δex51 and showed fewer indel byproducts [144]. Other advances have applied prime editing to rescue full-length survival motor neuron (*SMN*) gene expression [145] and to install genetic alleles protective against virus infection or cardiovascular disease [146]. In general, these studies indicate the remarkable potential of precise allele editing in treating diseases, especially for SNV therapy.

#### Treating complex diseases through multiple engineering

Growing evidence suggests that many human illnesses result from mutations at multiple genes or loci. Traditional gene manipulation or single gene editing becomes insufficient when dealing with these complex diseases. Base editors and prime editors mentioned above are precision genome editing tools, which can edit multiple mutations, enabling the treatment of polygenic diseases. Yuan et al. developed a drive-and-process strategy for multiplex base editing and multiplex prime editing, using tandem arrays of gRNAs, which has shown the ability for scalable multiplex desired edits [147]. Additionally, pegRNA pairs could be used to correct multiple mutational variants within the same gene [142], thus having the potential to cure heterogeneous patient populations. Early demonstrations of the Pompe disease model have shown the cross-correction of diseased cells [148]. These works establish the capacity to simultaneously program multiple precise DNA changes.

Furthermore, the discovery and understanding of gene editing will advance the applications of biotechnology in more complex diseases, such as cancer and aging, which are areas of popular concern. Human tumors exhibit recurrent evolution in genes encoding proteins involved in several pathways, including WNT, MAPK, TGF-b, TP53, and PI3K [149, 150]. Gene recorder tools, such as CHYRON (cell history recording by ordered insertion) [151] and CRISPRanibow [152], have been created to trace the emergence of subpopulations of tumor cells using complex “barcodes.” On the other hand, advanced multiplex gene editing could disrupt the development originating from cancer genome diversity, which is currently underway for lung, prostate, and renal cell cancer [153]. Other studies have shown that multiple editing could reverse aging, employing two strategies: (i) cell-selves, by editing longevity genes such as *FGF21*, *Stgf-βR2*, and *αKlotho* [154]; (ii) surrounding cells by regulating cytokines such as inflammatory factors and Yamanaka factors [155], which have been demonstrated in mice to improve or completely reverse age-related obesity, type II diabetes, heart failure [156], and successfully restore retinal nerve cells to light [157]. More clinical trials, particularly involving mitral valve disease, are being conducted in dogs. We anticipate that the combination of these emerging gene editing tools will continue to provide promising avenues for complex disease therapeutics.

### Therapeutic nucleic acid drugs

Therapeutic nucleic acid is a new generation of pharmaceutical technology after protein drugs, and it is becoming a promising candidate in the biomedical domain [158]. Therapeutic nucleic acids are modified RNA or DNA with different functions, mainly playing roles at the gene level for the treatment of diseases. Conventional treatments target proteins rather than the underlying causes, resulting in generally transient therapeutic effects. In contrast, nucleic acid therapeutics directly act on the pathogenic target gene or mRNA, achieving long-lasting or even curative effects [159]. As elaborated in this review, nucleic acid-based therapeutics have been adapted to develop a broad array of applications, including cancer, infectious disease, neuromuscular disease, lung disorders, and ocular disease therapies. Based on the mechanism of action, here we highlight several applications of programmable nucleic acids and DNA-/RNA-based vaccines (Fig. 5C).

#### Programmable nucleic acids

Programmable nucleic acids refer to DNA or RNA molecules that are used for gene silencing therapy at the transcriptional level. Here we primarily focus on therapeutically relevant RNA, which complementarily pairs and degrades target mRNA, thus inhibiting translation. Several RNA-based therapeutic modalities have been developed to manage and treat diseases, including microRNA (miRNA) [160], small interfering RNA (siRNA) [161], and antisense oligonucleotide (ASO) [162].

Both miRNA and siRNA play a role through RNA interference (RNAi), which degrades mRNA specifically, leading to compelling gene silencing [163]. These advanced therapies, with lower side effects, wider location, and higher selectivity, are expected to become an essential approach in treating a variety of viral-infectious, cardiovascular diseases, hemato-oncological cancers, and neurodegenerative conditions [164]. siRNA, short double-stranded RNA consisting of 19–25 bp, has the antisense-strand loaded into the RNA-induced silencing complex (RISC), leading to the degradation of the target mRNA transcript and preventing translation. Therefore, any pathogenic gene could be a potential candidate, as verified by numerous siRNA clinical trials. Only a few examples could be cited of these trials, while the current major challenges are related to their protection and efficient delivery *in vivo*. Hossain et al. previously developed a CpG-signal transducer and activator of transcription (*STAT3*) siRNA conjugates, which provided a promising solution for cancer cell growth, as demonstrated by the CAS3/SS3 clinical trial [165]. However, clinical application is still limited by several carrier-induced restrictions. Recent research explored F-based-modified siRNA (F-siRNA), showing improved biostability and specific accumulation in tumor tissue [166]. Other siRNA formulations, such as lipid nanoparticle (LNP) [167, 168] and 1,2-dioleoyl-sn-glycero-3-phosphatidylcholine (DOPC) neutral liposome [169], are in the process of approval. Patisiran, the first approved siRNA drug, was awarded by the FDA in 2018 [170], and there are currently five siRNA drugs available. Compared with siRNA, miRNA is much more complex. The crucial function of miRNA is to negatively regulate the target mRNA [171], and they are typically around 22 nt in length. One miRNA could regulate multiple mRNAs, and one mRNA could be regulated by multiple miRNAs. Due to its close relationship with diseases, miRNA has been regarded as a core element. The present development of miRNA drugs focuses on miRNA mimics and miRNA inhibitors, but there are no commercialized drugs currently.

ASO, single-stranded oligonucleotides ranging from 13 to 25 nt in length, complementary bind to mRNA, preventing its translation and inhibiting the expression of disease-related genes [172]. More than 10 ASO drugs have been listed in succession and obtained good results, such as Defibrotide [159], Fomivirsen [173], Nusinersen [174], and Eteplirsen [175]. Among them, Eteplirsen was the first drug approved to treat DMD, and the drug’s development soon expanded to various disease fields, including nervous system diseases, and cardiovascular and metabolic diseases [176]. Recently, Tofersen, the first ASO drug for amyotrophic lateral sclerosis (ALS) was approved. It binds to the mRNA encoding superoxide dismutase1 (SOD1), thus reducing the production of the mutant protein.

#### DNA-/RNA-based vaccines

Nucleic acid vaccines employ DNA or RNA plasmids as antigen precursors that encode viral antigens and are translated into proteins by host cells. These vaccines can elicit cellular and humoral immunity and have been widely used in the swift global response to the COVID-19 pandemic. Synthetic biology and biochemical approaches have been used to enhance these nucleic acid vaccines, allowing for rapid design and simplicity of production [4]. This advantage has been particularly highlighted during the COVID-19 crisis, as no existing licensed nucleic acid vaccines have been used before [177]. Building on this momentum, clinical progress has been extended to include vaccines against influenza, human immunodeficiency virus (HIV), and cancer.

DNA-based vaccines were initially developed due to their better stability but raised concerns about undesirable genomic integration. RNA-based vaccines, on the other hand, do not have the integration problem but face challenges related to rapid degradation by ubiquitous nucleases. Therefore, both nucleic acid vaccines rely on robust delivery tools to protect and enhance expression. For instance, the ZyCoV-D vaccine [178], a DNA-based vaccine encoding SARS-CoV-2 proteins, showed no severe related side effects and had been approved in India. There are currently 11 candidate DNA vaccines under clinical trials.

RNA-based vaccines encode antigen genomes and can be directly translated into the cytoplasm of host cells. The two approved mRNA vaccines, Pfizer-BioNTech’s BNT162b2 [179] and Moderna’s mRNA-1273 [180], are vaccines with chemically modified uridine bases and are the only two approved products among seven phase III clinical SARS-CoV-2 vaccines. Moreover, the rapid development of therapeutic cancer vaccines based on mRNA has showcased immense potential. In 2021, BioNTech received FDA Fast Track designation for BNT111 [181], a mix of four melanoma-related antigens (NY-ESO-1, tyrosinase, MAGE A3, and TPTE), and the combination of mRNA vaccines with checkpoint inhibitors is showing promising potential for cancer treatment. In the latest research, BNT122 [182], an individualized new antigen-specific cancer vaccine developed by BioNTech, combined with anti-PD-L1 antibody atezolizumab and chemotherapy, significantly delayed the recurrence time of pancreatic patients and further demonstrated their potential.

### Cell therapy (CAR-T)

Cell therapy involves the use of modified or non-modified cells cultured *in vitro* and then transfused to work *in vivo*. Chimeric antigen receptor (CAR) T-cell therapy is one of the most successful cases of engineered cell therapies and is now an established example of clinically approved treatments, demonstrating a powerful modality for human diseases. CARs comprise of scFv, CD8α transmembrane, CD3ζ, and CD28 or 4-1BB co-stimulatory domains [183]. Activation of stimulatory and co-stimulatory domains is promoted by attachment of a target antigen to the scFv, leading to T-cell proliferation and target cell death. T cells equipped with the positioning device “CAR” can specifically identify tumor cells and release various effector factors, demonstrating potent anti-cancer cytotoxicity. There is increasing evidence that CAR-T cells have strong anti-tumor therapeutic potential based on clinical or preclinical trials targeting tumors such as large B-cell lymphoma (DLBCL), multiple myeloma, and acute lymphoblastic leukemia. However, many pressing challenges remain to be addressed, particularly in the context of solid tumors. Advanced therapeutic cell designs with enhanced precision and control are necessary to overcome these problems.

Synthetic biology offers new possibilities for designing engineered chimeric T cells with enhanced control, flexibility, and specificity in cellular therapies [184, 185]. One of the unique advantages of CAR-T therapies is the ability to implement genetic control circuits. Genetic logic circuits, foundational decision systems in synthetic biology, such as AND, OR, and NOT [186], when further amplified into an artificial synthesis program that recognizes specific signals and performs specific functions, enable the creation of CAR-T cells with greater precision and accuracy. In this perspective, we outline two advanced strategies: autonomous control and external control (Fig. 5D). Immune cells engineered to express autonomous circuits can bind signals from engineered immune cells or native environments [187, 188], including intracellular cell states, antigens, and the tumor microenvironment. Another key approach is to design cell therapies with different signals from external reagents, such as lights, ultrasound, and small molecules [187].

Autonomous control signals can be deployed in combination to address complex cancer conditions. Here, we highlight two advanced logic circuits: the split, universal, programmable CARs (SUPRA CAR) system, and the Colocalization-dependent Latching Orthogonal Cage-Key pRoteins (Co-LOCKR) system. The core design principle of these multi-input circuits is to create a universal CAR platform and docking adapters. Each signal pathway corresponds to a unique CAR, and modulating the balance of multiple signals require varying the number of functional CARs. A split universal CAR platform is one of the most direct ways to modulate CAR signaling strength. A versatile example is the SUPRA CAR system, which consists of leucine-zipper universal CAR receptors (zipCAR) and zipFv with an scFv fused to a cognate leucine zipper that can orthogonally bind [188]. The extended dually inducible SUPRA CAR system demonstrates the multiple controls to regulate different T-cell subsets independently. Another example is the Co-LOCKR system [189], which comprises a CAR and two adaptor domains: Cage and Key. Only cells with both antigens can colocalize Cage and Key, leading to the exposure of the Cage domain and subsequent activation of the CAR.

Moreover, exogenous control capable of directing tumor features has also been desirable for relevant clinical therapies. The hypoxia-sensing CAR-T system is designed to selectively express within solid tumors, which are characterized by low oxygen supply, facilitating the unlimited expansion of CAR-T cells in the target conditions and demonstrating anti-tumor efficacy without off-tumor toxicity [190]. Furthermore, other solutions with exogenous gene control circuits provide credible security, acting as an ON or OFF switch, and can be further combined with autonomous circuits. For example, the versatile protease regulatable CAR (VIPER CAR) system comprises small molecular induced ON and OFF switch CAR circuits with an NS3 protease domain, and when combined with SURPA CAR, it results in an ON-OFF VIPER CAR that not only changes zipFv but also shuts off the safety switch when needed [184].

Five malignancy therapies approved by the FDA have shown promising potency and clinical anticancer cytotoxicity [191]. However, several additional challenges need to be addressed for more circuit systems to become clinically translatable therapeutics. Balancing over-activation and off-target activity will further improve curative effects and accuracy. Increasing the number of CAR-T circuits with high specificity, effectiveness, and safety should be powerful for treating more stubborn diseases.

### Virus-based disease treatment

#### Virus vaccines

Vaccines are one of the most effective components to prevent and control numerous infectious diseases, as evidenced by the COVID-19 pandemic. In addition to the DNA-/RNA-based vaccine mentioned above, virus-based vaccines are more mature and have been clinically proven popular measures, including inactivated, live attenuated, and virus-like particles. Inactivated vaccines are easily generated but provide short-term protection. Here, we particularly focus on synthetic biology for generating effective attenuated live virus and virus-like particles (VLPs) vaccines (Fig. 5E). Live attenuated vaccines are weakened pathogens that mimic natural infections in the host, inducing related immunity, while considerably reducing harmfulness. Serial passaging has been applied to develop attenuated virulence vaccines, but advances in synthetic biology have allowed to creation of attenuated viruses using under-represented codons and codon pairs without requiring detailed knowledge of viral function [183]. This technology has generated live attenuated poliovirus [192], influenza [193], SARS-CoV-2 [177], Ebola Zaire [194], and smallpox [195] vaccines. VLPs vaccines, as the name suggests, are macromolecular assemblies that mimic native viral morphology, encoding at least two original viral structural components but cannot replicated [196]. This technique has been used to explore human papillomavirus, ZIKA, and SARS-CoV-2 viruses [197]. Furthermore, it has facilitated vaccine development through the use of advanced platforms such as the split-virus-genome (SVG) system, which expresses reconstituted virus particles (rVPs) [198]. The VLPs platform with alternative vaccine designs and epitope prediction, leveraging data-rich disciplines such as machine learning and AI, holds great promise for the future [199].

#### Viral vector for drug/gene delivery

Advancements in gene therapy have led to the emergence of viral vectors for delivering therapeutic proteins and gene editing agents. These include mAbs (antibodies), anticoagulants, blood factors, enzymes, growth factors, hormones, and engineered proteins [200]. Therapeutic gene editing agents such as deliver base editors [201] and Cas9 ribonucleoproteins [202], have also contributed to the development of viral vectors. The most commonly used viral vectors in research and clinical trials are retroviral, adenoviral, and adeno-associated viral (AAV), herpes simplex viral (HSV), and lentiviral (Fig. 5E). Natural viruses have evolved efficient mechanisms to enter host cells and replicate [203], and these structural characteristics can be exploited to deliver therapeutic genes into target cells. The field of synthetic biology, which applies engineering principles to design biological systems using interchangeable parts, has played a pivotal role in this exciting area. Over the past two decades, more than 20 viral vector therapies have been approved [204], with a focus on treating cancer, monogenic, and infectious diseases. Among these, AAV vectors have gained widespread use for *in vivo* gene vector applications. AAV is a nonpathogenic parvovirus with a 4.7 kb DNA genome enclosed in a nonenveloped icosahedral capsid [205]. Notably, the first approved cancer viral gene therapy, GENDICINE, is based on human Ad5, where the E1 gene was replaced with the tumor suppressor gene *P53* [206]. GENDICINE has been clinically studied for various cancers, including lung, breast, ovarian, and liver cancer [207]. Additionally, all four approved viral vector vaccines against SARS-CoV-2 (Ad26.COV2.S, ChAdOx1-nCov-19, Gam-COVID-Vac, and AD5-nCoV) are based on recombinant adenoviral vectors. Clinical trials have demonstrated their remarkable potential in treating diverse diseases including hematological diseases such as hemophilia A and B, musculoskeletal disorders such as DMD, and neurological diseases such as spinal muscular atrophy [208]. These viral vectors can be further improved through directed evolution or structural design and have given rise to novel tools with key advantages, such as eVLP (engineered virus-like particles) [202]. Furthermore, viral vectors can be combined with CAR-T and have shown tremendous success in treatment [209].

Although viral vectors have been increasingly successful in clinical trials for drug and gene delivery, there are still such challenges. The widespread concerns are immune responses, production costs, and precise regulation. It is crucial to understand the interactions between viral vectors and the immune system, as severe immune reactions induced by *in vivo* delivery can lead to numerous complications, in some cases resulting in death [210]. This variability is vector-dependent. For adenoviral vectors, deciphering how to manage adverse immunological responses that lead to severe side effects is crucial, while lentiviral vectors, on the other hand, have very favorable immunogenicity profiles. Production costs are another significant obstacle that must be overcome for clinical application. For instance, in the case of AAV vectors, small-scale manufacturing under current good manufacturing practices (GMP) is a limitation, affecting the speed of gene therapy development and manufacturing [211]. The rapid modification of gene editing, targeting specific genes to specific tissues or cells for expression in specific timeframes, shows encouraging prospects, although more research is needed to avoid off-target effects, achieving strictly regulated delivery expression. Overall, due to their limited adverse reactions and favorable clinical data, these viral vectors appear to be favorable choices for future medicine.

#### Phage-therapy

In the last 5 years, phage therapy has experienced a resurgence due to the increasing antimicrobial resistance (AMR) problem [212, 213]. Phages, which are the most common type of double-stranded DNA (dsDNA) tailed phages, infect bacterial cells by attaching their tail tip to the bacterial cell wall and injecting their genome into the capsid (head). These phages can be classified as lytic or temperate (Fig. 5E). Lytic phages undergo a developmental program in the early stage, involving gene expression and genome replication, followed by a lyric program in the late stage, which assembles genes and packages viral particles. On the other hand, temperate phages can either undergo lytic growth or establish lysogeny, depending on various factors. Phage therapy, either used alone or in combination with antibiotics, has been described for the treatment of infections [214]. Recent advances in phage design, leveraging genetic logic circuits from synthetic biology, have enabled the incorporation of artificial input and output systems, such as lambda Red and Rac RecE/RecT homologous recombination and genome synthesis [215]. This has led to enhanced therapeutic properties, safety features, and an expanded host range for phage therapy. Since 2020, 29 clinical trials involving phage therapies have been initiated [212], and further research aims to carefully evaluate their potential for combating AMR. Engineered T3 phage tail fiber mutagenesis has resulted in synthetic “phagebodies,” with diversified host ranges, enhancing the efficacy of therapeutic phages [216]. Moreover, an engineered lambda phage that incorporates a CRISPR-Cas3 system has demonstrated high specificity and efficiency in eliminating enterohemorrhagic *E. coli* (EHEC) [217]. Other efforts, such as CRISPR/Cas-based iterative phage genome reduction (CiPGr), provide valuable insights into whether genome reduction can lead to phages with new infectious properties, paving the way for future genome synthesis research. In addition to therapeutic-engineered phages, phages can also be engineered or modulated to deliver Cas nucleases as antimicrobial payloads [215]. Recent studies combining phage delivery with CRISPR-Cas3 antimicrobials have demonstrated improved therapeutic effects in a mouse model of *Clostridioides difficile* infection. The use of machine learning and AI holds promise in tapping into the vast untapped potential of phage therapy, enabling better prediction and evaluation of phage treatments for multi-resistant diseases in future.

### Bacteria therapy

#### Active bacterial drugs

In addition to phages, bacteria have emerged as a promising therapeutic platform, offering extraordinary advantages such as their ability to self-propel inaccessible tissues, precise spatiotemporal control, and immune activation or metabolism with individual proliferation [218]. These features can be further harnessed to develop more effective therapies, as synthetic biology allows for the re-editing of diversified bacteria to provide potential treatments for various illnesses, including metabolic, gastrointestinal, and oncological diseases [219]. Designer bacteria involve modified realization of inherent structures or functions, and subtractive implementation of intrinsic pathogenicity or behaviors [220]. Engineered microbes can be defined as generalized gene circuits, capable of performing artificial-set input, operation, and output, and may be employed as targeted vehicles for specific pathogens or delivery vehicles of therapeutic molecules (Fig. 5F).

Engineered therapeutic bacteria hold great promise for future tumor therapy, primarily due to their unique colonization and utility in hypoxic and immunosuppressive tumor microenvironments (TMEs). These engineered bacteria effectively localize payloads intracellularly or extracellularly, which can be released by secretion, diffusion, or lysis mechanisms [218]. One strategy to improve tumor localization involves engineering bacteria to display tumor-targeting moieties. For example, an attenuated *S. Typhimurium* strain carrying a sandwich fused-gene expression cassette encoding outer membrane protein A (OmpA) bearing the RGD-4C (ACDCRGDCFCG, cyclic arginine-glucine-aspartic acid) peptide demonstrated strong initial targeting and subsequent proliferation within tumors, as demonstrated by adhesion and competition assays [221]. Another approach aims to enhance the tropism of engineered bacteria by coupling bacterial growth with genetic circuits that respond to essential factors such as oxygen, pH, or lactate. The integration of “AND gates,” which couples hypoxia and lactate biosensors, has shown the broad applicability of multiplex genetic circuits in enhancing the localization of engineered bacteria [222]. Certain therapeutic strategies necessitate extracellular delivery methods, such as the use of engineered commensal *E. coli* tool PROT_3_EcT [223], which can direct the secretion of proteins into the surroundings. Live bacteria have increasingly been explored for *in vivo* anti-tumor immunogenicity, as studies have demonstrated that tumor colonizing bacteria can increase the levels of IL-1β, TNF-α, and IFN-γ, thereby stimulating NK and T cells, indicating their potential to eliminate cancer cells [224]. A novel example involves using attenuated *S.typhimurium* to express chemokine CCL21, resulting in significant inhibition of primary tumor growth [225]. Moreover, engineered commensal bacteria, such as modular programmable genetic circuits in *Bacteroides thetaiotaomicron* that respond to external signals [177], or engineered gut-associated bacteria as real-time diagnosing biomarkers [226], can be designed as long-lasting therapeutic platforms that interact with human tissues.

#### Bacteroides therapy

Live bacteria can be synergistically integrated with anti-cancer drugs and nanomaterials to increase the targeted delivery and accumulation of the latter within tumors. Several types of bacterial membrane vesicles (BMVs) (Fig. 5F), have been used as vaccines or vaccine adjuvants against viral infections and cancer. These vesicles include membrane vesicles (MVs) from gram-positive bacteria, outer membrane vesicles (OMVs) from gram-negative bacteria, double-layered membrane vesicles (DMVs), and protoplast-derived nanovesicles (PDNVs) [227]. For example, OMVs have been used as antibiotic delivery vehicles, while DMVs have been explored as phosphodiesterase delivery systems, offering diverse therapeutic applications, including cancer treatment, immunotherapy, and infection management [228]. Moreover, engineered bacterial communities show promise in smarter tutor treatment, primarily through quorum sensing (QS) mechanisms [229]. For instance, enteric bacterium, *E. coli*, which increases intestinal autoinducer-2 (AI-2), a QS signal, can alter the composition of gut microbiota, known to significantly influence human health [230]. Additionally, a novel synchronized lysis circuit (SLC) has been developed, enabling bacteria to lyse when reaching a certain population density, followed by the multiplication of a few survivors in the next pulsatile cycle [231]. The integration of multiple bacteria into engineered consortia with intricate metabolic interactions can further enable complex cascade reactions for diverse biomedical strategies, presenting a promising direction for the future development of engineered bacteria therapies [232].

### Other applications

#### Engineering hosts for organ transplantation

Germline engineering has been devoted to developing organs, tissues, and cells for human transplantation. The goal is not only to address organ defects but also to multi-edit organs to outperform natural human organs (Fig. 5G). In previous studies, over 40 genetic modifications have been attempted on pigs, either individually or in combination, to mitigate PERV (porcine endogenous retroviruses) transmission [233, 234] and molecular incompatibility [235]. In a recent clinical operation, a 10-gene-edited pig heart was transplanted into a human who was suffering from a fatal heart attack, however, two months later, the patient tragically succumbed to porcine cytomegalovirus infection [236].

#### Organ-on-chip

While traditional two-dimensional (2D) *in vitro* models and animal models, such as cell cultures, have significantly contributed to studying pharmacodynamics and pharmacokinetics, they fail to replicate the complexity of the microenvironment. In contrast, three-dimensional (3D) models more precisely mimic the spatial characteristics and microenvironment of physiological pathologies, encompassing both scaffold-free, and scaffold-based cultures. The organ-on-chip system is a scaffold-based biomimetic 3D technology, centered around a microfluidic chip, constructing an *in vitro* environment encompassing various live cell types, tissue interfaces, biological fluids, and mechanical force stimuli, mimicking the microenvironmental factors of *in vivo* organs. This reproduction of *in vivo* micro physiological systems offers a novel option for the study of diseases and drug screening. Organ chips present the opportunity to replicate the complex structures and physiological functions of major human functional units, holding the potential to reduce the high failure rate in current drug development pipelines [237]. Amidst the COVID-19 pandemic, several airway and alveoli organ-on-chips models have been developed to study SARS-CoV-2 kinetics, tropism, and host responses [238]. A micro-engineered human lung chip was designed to model alveolar infection by native SARS-CoV-2 and evaluate drug efficacy [239], providing insights into immune responses and cell–cell interactions. These organ-on chips have shown their potential to accelerate the identification of existing approved drugs. For instance, high-level clinical mimicry airway chips with different membrane chemistry and larger pores were able to recapitulate organ-level pathophysiology and cell differentiation, leading to the identification of multiple approved drugs, which could serve as therapeutics or prophylactic against SARS-CoV-2, including oseltamivir, hydroxychloroquine, and amodiaquine [240]. A recent study reported a micro-engineered 3D model of epithelial–mesenchymal transition (EMT) that evaluated the efficacy of 12 drugs during cancer progression [241]. Additionally, 3D blood-brain organ-on chip models, have exhibited robust and realistic results, providing a versatile and valuable platform for neuroscientific research, drug testing, and pharmaceutical development [242].

## Conclusion and future perspectives

Over the past decade, synthetic biology has made remarkable progress in medical practice. In this review, we have highlighted the transformative impact of synthetic biology on medicine, particularly the advanced strategies and designs that have revolutionized the way biologists approach various aspects. Specifically, we have discussed the acceleration of medical production, the facilitation of disease diagnosis, and the revolution in disease treatment. We have covered the current hot spot and promising focused medical applications of synthetic biology, including cell factories for drug production, bio-catalysis, new drug discovery, gene circuits for disease surveillance, gene editing and genome recoding, and live probiotic therapeutics. Certain applications, such as commercial cell factories and CAR-T therapy, have matured significantly and garnered attention from enthusiastic investors, while others, such as nucleic acid vaccines and viral vector delivery, have already made significant contributions to global epidemic disease management. The inception of synthetic biology in medical applications is now becoming unstoppable.

The growing biomedical aspirations, once considered unattainable, are now being pursued through the new frontier of synthetic biology. The evolution is transitioning from pharmaceutical or therapeutic nucleic acids to modular gene circuits and further into artificial living cell systems, and eventually, it may extend to organs and germlines. The next generation of treatment strategies appears to be heading toward increasingly complex genetic logic. Although synthetic biology is propelled by the central principles of molecular biology, our understanding of life remains a lifelong study, and we are still at an early stage of comprehending the mechanisms involved. For example, the general systems for controlling synthesis and endogenous protein have yet to be fully reported. As synthetic systems become larger and more complex, their interaction with endogenous systems becomes more obvious [243]. The current artificial system imposes different but considerable physiological burdens on the host, raising the crucial concern of how to seamlessly integrate these modules into organism systems.

One of the long-term scientific pursuits of synthetic biology appears to be the creation of biological intelligence. Significant progress has already been made, with precedents set in the synthesis and assembly of recoded *E. coli* fragments, the complete small genome of *Mycobacterium genitalium*, and the full set of yeast chromosomes (Sc2.0) [70]. Recent studies, such as artificial orthorhmbic systems [16], DNA storage [244], and genome writing [245], have further demonstrated the possibilities that suggest this goal is attainable. These achievements open the door for *de novo* design, innovation, and even the transcendence of life itself, revolutionizing medicine. While barriers related to genome-wide synthesis and sequencing technologies continue to decrease in cost, the depth of knowledge and mechanistic insights necessary for completely synthetic biological controls still lag. Achieving this requires a deeper understanding of how organisms function at the molecular and individual levels. Several researchers are working on building such networks and describing protobiology at the systems level, moving away from reductionism to focus on components and pathways, followed by upward re-engineering [246].

Another important avenue of experimentation involves further crossover with interdisciplinary integration [247], including computer-aided design (CAD) [248], biological logic computation [249, 250], and AI. Notably, the integration of AI has led to significant breakthroughs, not only in predicting natural protein structures but also in designing entirely new proteins. Machine-learning algorithms, such as AlphaFold2 [251] and RoseTTAFold [252], have demonstrated their potential in predicting protein structures and have been used to screen millions of protein sequences. CAD has also shown strength by designing proteins from scratch through computer assistance, resulting in innovative functions and structures. The latest advances have allowed designing backward from a desired function to an appropriate structure to a suitable amino acid sequence. This means that any random amino acid sequence can be given, and an entirely “hallucinated” protein can be folded [253]. The future holds great potential for AI and protein *de novo* design in various medical applications. Furthermore, the increasing multi-disciplinary integration with synthetic biology will undoubtedly usher in a new era of “synbio innovate medicine.”

Synthetic biology is increasingly gaining emphasis in medical applications, with many efforts focusing on integrated *in vivo* monitoring treatment platforms and controlled targeted drug delivery. Regardless of the specific application, synthetic biology showcases innovative technological collaborations between academia and industry. However, considerable challenges lie ahead, particularly concerning safety, bioethics, and legal considerations. As medical therapeutics ultimately aim to address human health problems, addressing these shortages and bottlenecks becomes vital.

This review only scratches the surface of the vast possibilities enabled by synthetic biology. Given the extraordinary progress thus far and the numerous ongoing efforts in medical applications, we anticipate that the black box of species structure and function will be opened up in the future. This will lead to a leap from principle-set products to intelligent designs within biology. Overall, synthetic biology marks the beginning of a new era in the medical field.
